# Volumetric Measurement of Peripapillary Hyperreflective Ovoid Masslike Structures in Patients with Optic Disc Drusen

**DOI:** 10.1016/j.xops.2021.100096

**Published:** 2021-12-21

**Authors:** Morten Jørgensen, Lasse Malmqvist, Alexander E. Hansen, J. Alexander Fraser, Steffen Hamann

**Affiliations:** 1 Department of Ophthalmology, Rigshospitalet, University of Copenhagen, Glostrup, Denmark; 2 Departments of Clinical Neurological Sciences and Ophthalmology, Western University, London, Canada

**Keywords:** Optic disc drusen, OCT, Optic nerve anatomy, Optic nerve head drusen, Peripapillary hyperreflective ovoid masslike structure, PHOMS, Volumetric measurement, BMO, Bruch’s membrane opening, EDI, enhanced depth imaging, IQR, interquartile range, NAAION, nonarteritic anterior ischemic optic neuropathy, ODD, optic disc drusen, PHOMS, peripapillary hyperreflective ovoid masslike structure(s)

## Abstract

**Purpose:**

To develop a method to determine the volume of peripapillary hyperreflective ovoid masslike structures (PHOMS) and to examine the correlation between PHOMS and anatomic optic nerve head characteristics in a large cohort of patients with optic disc drusen (ODD).

**Design:**

Retrospective, observational study of patients with ODD.

**Participants:**

Patients with ODD seen in a 3-year period.

**Methods:**

We determined the prevalence of PHOMS. We then developed a method to calculate the volume of PHOMS and measured this in all patients where radial scans on OCT were available. We analyzed the correlation between PHOMS volume and patient age, size of Bruch’s membrane opening (BMO), ODD visibility, and anatomic location of ODD in the optic nerve.

**Main Outcome Measures:**

Prevalence and characteristics of PHOMS in patients with ODD.

**Results:**

In 247 (77%) eyes with ODD, PHOMS were found. Among these, 80% were in the first decade of life, 87% were in the second decade, 89% were in the third decade, 85% were in the fourth decade, 74% were in the fifth decade, 73% were in the sixth decade, 58% were in the seventh decade, 40% were in the eighth decade, and 0% were in the ninth decade. The ophthalmoscopic visibility of ODD increased with age. The volume of PHOMS decreased with age, but with no correlation to the size of BMO. The median volume of PHOMS was 0.27 mm^3^ (interquartile range [IQR], 0.13–0.49 mm^3^). Predominantly, PHOMS were observed in the nasal peripapillary area (87.5% nasal, 78.5% superior, 67% inferior, and 63.5% temporal).

**Conclusions:**

In patients with ODD, PHOMS are seen frequently, with the highest prevalence in younger individuals. The volume of PHOMS decreases with age, and PHOMS are seen more frequently in patients with superficial ODD.

The development and subsequent application in practice of enhanced depth imaging (EDI) OCT has improved the imaging of the optic nerve head and peripapillary region tremendously^1^ and has led to the discovery and characterization of a new entity: the peripapillary hyperreflective ovoid masslike structure(s) (PHOMS). Peripapillary hyperreflective ovoid masslike structures are located above Bruch’s membrane, extending circumferentially around the edge of Bruch’s membrane opening (BMO) in a complete or partial torus (donut shape).^2^ Peripapillary hyperreflective ovoid masslike structures are common in patients with optic disc drusen (ODD),^3^, ^4^, ^5^, ^6^ patients with anomalous optic discs such as myopic or tilted discs,^7^^,^^8^ and patients with optic disc edema of any kind.^4^^,^^9^, ^10^, ^11^ The common denominator among these conditions seems to be the peripapillary axonal distension, which takes place as a consequence of axoplasmic stasis or peripapillary axonal bending or squeezing in the scleral canal^12^^,^^13^; however, the exact pathophysiologic features leading to PHOMS development remain unknown, and the secondary consequences of PHOMS for optic nerve function have not yet been investigated. Because PHOMS consist mainly of retinal nerve fibers, and because peripapillary retinal nerve fiber layer thickness is known to decrease with age,^14^ the hypothesis is that PHOMS volume decreases over time as a consequence of normal aging. It is unknown whether local anatomic characteristics within the optic nerve head, such as superficial or deep ODD, have any correlation with PHOMS volume and whether such a correlation decreases with age. The purpose of the present study was to calculate the volume of PHOMS and to examine the correlation between PHOMS volume and underlying anatomic optic nerve head characteristics in a large cohort of patients with ODD.

## Methods

### Study Population

We performed a retrospective observational study of patients with ODD. We included all patients with an OCT-verified diagnosis of ODD seen in our clinic from November 2017 through November 2020. Patients of all ages were included. We excluded eyes without ODD and eyes with ODD-associated anterior ischemic optic neuropathy or other ODD-associated complications. The handling and storage of data were approved by the Knowledge Center for Data Reviews of the Capital Region of Denmark (identifier, RH-2017-316). All procedures adhered to the tenets of the Declaration of Helsinki. All participants or their parents or guardians provided informed consent.

### Data Acquisition

All patients underwent OCT (Spectralis HRA + OCT; Heidelberg Engineering). Most patients underwent dense optic nerve head scan with EDI OCT with 30 μm between each B-scan (97 scans) and radial scans with either 6 or 24 scans centered in the center of the optic disc, according to the Optic Disc Drusen Studies Consortium guidelines.^4^

### Image Analysis

The OCT scans were independently assessed by 2 masked investigators (M.J. and A.E.H.) for the presence of concurrent ODD and PHOMS. If radial scans were available, the diameter of BMO was measured as an average of 6 radial scans. Whether ODD was visible on the corresponding fundus photography was noted. Optic disc drusen not visible on fundus photography were labeled as buried ODD. One investigator (M.J.), using the dense optic nerve head EDI OCT scans, noted for each eye whether most ODD were above or below BMO, which we labeled as superficial or deep, respectively. If any doubt existed regarding whether to classify as superficial or deep, 2 other masked investigators (L.M. and S.H.) independently reviewed and classified these scans. In case of classification disagreements in this review, a collective review (L.M. and S.H.) finally ensured consensus. For clarity, it should be noted that the photographic classifications of visible versus buried and the EDI OCT classifications of superficial versus deep are distinct and independent classification schemes.

**Figure 1:**
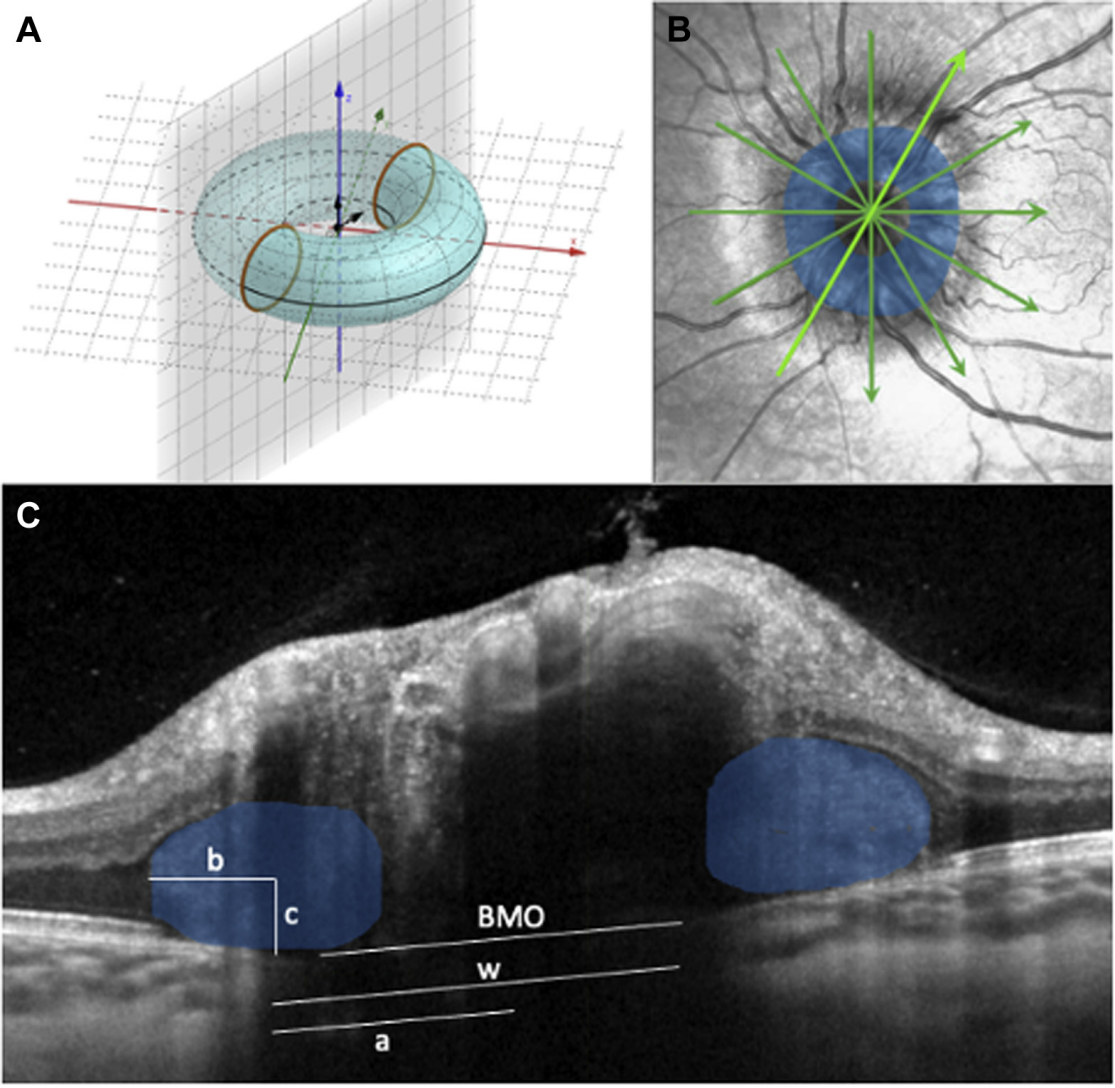
Volume measurement of peripapillary hyperreflective ovoid masslike structure(s) (PHOMS). **A**, **B**, A PHOMS encircles the optic disc in 3 dimensions, like a torus. **C**, In each of the 6 radial OCT scans, it was noted on both side of the Bruch’s membrane opening (BMO) if a PHOMS fragment (blue oval) was present or not. If 2 PHOMS fragments were present in all 6 scans, they were counted as part of 1 large PHOMS extending all the way around the disc like a torus. In each radial scan, for each PHOMS fragment, we measured *b* = PHOMS fragment cross-sectional width divided by 2, *c* = PHOMS fragment cross-sectional height divided by 2, *w* = the distance between the far edge of BMO to the intersection of *b* and *c*, and *a* = *w* – ½BMO. The minor radii of the torus are *b* and *c*; the major radius of the torus is *a*.

To calculate PHOMS volume, each PHOMS was modelled as a complete or partial torus whose major and minor radii were measured and averaged over all 6 to 24 radial OCT sections. Individual OCT cross-sections through a 3-dimensional PHOMS depict 2 2-dimensional ovals on either side of the BMO, which we term *PHOMS fragments* (Fig 1). The geometry of a torus is such that the measurement of the height, width, and distance from the center of BMO of these 2 oval PHOMS fragments is sufficient to calculate the volume of the torus (described below). Because a PHOMS may be an incomplete torus (e.g., a C shape instead of an O shape), PHOMS fragments may not be seen, in which case their height and width were recorded as 0. Whether PHOMS fragments were present in the nasal, temporal, superior, or inferior peripapillary space, or a combination thereof, was also recorded.

### Peripapillary Hyperreflective Ovoid Masslike Structure Volume Measurement

In each of the 6 to 24 radial OCT sections, it was noted on each side of the BMO whether a PHOMS fragment was present. If 2 PHOMS fragments were present in all 6 scans, they were counted as part of 1 large toroidal PHOMS extending 360° around the disc (Fig 1).

In each radial OCT section, and for each PHOMS fragment, we measured 4 parameters: *b*, which was the horizontal cross-sectional radius (width divided by 2); *c*, which was the vertical cross-sectional radius (height divided by 2); *w*, which was the distance between the far edge of BMO to the center of the PHOMS fragment (the intersection of *b* and *c*); and BMO, which was the width (diameter) of the BMO. From these parameters, we were able to calculate: *a*, which was a major radius of the torus, equivalent to *w* – ½BMO. The averages of *a*, *b*, and *c* were calculated across all PHOMS fragments. The volume of the PHOMS could then be calculated using the geometric formula for the volume of a torus:

$$\text{Total PHOMS volume}=\;2\pi^{2}abc,$$

where *a*, *b*, and *c* are the arithmetic mean values in millimeters.

### Statistical Analysis

For statistical analysis, data were analyzed using RStudio software version 1.3.1093 (RStudio, PBC).

## Results

**Figure 2:**
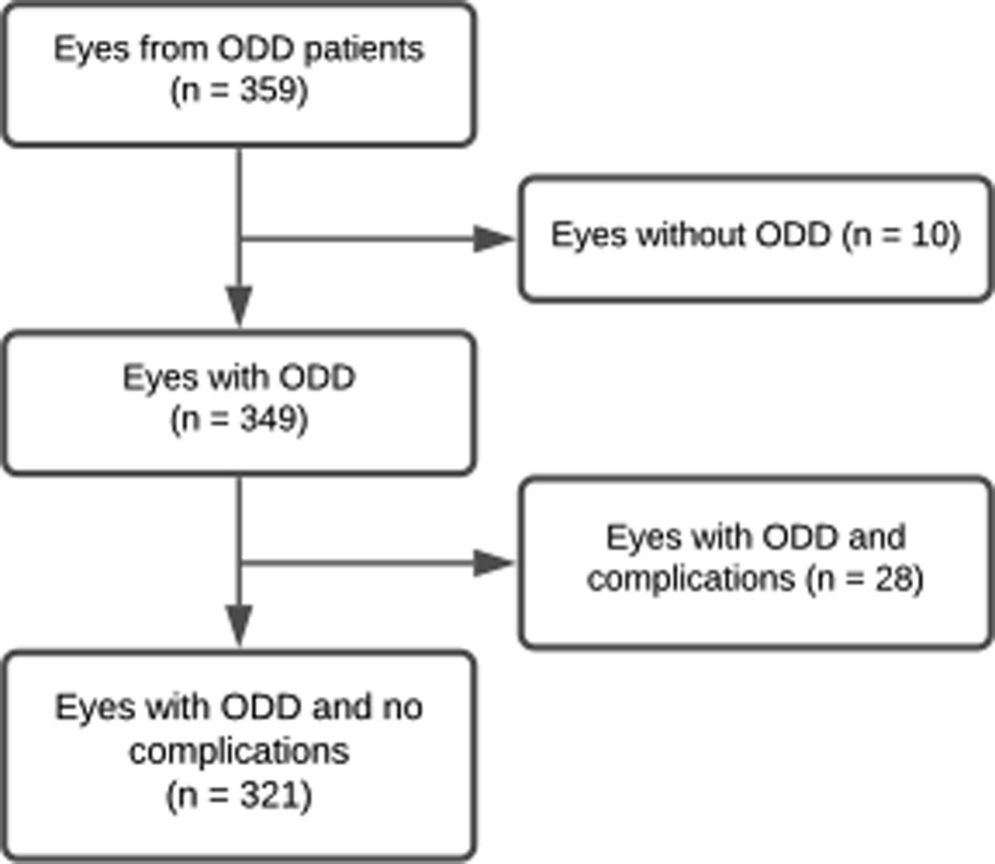
Flowchart showing the inclusion and exclusion of patients. ODD = optic disc drusen.

We included 359 eyes from 180 patients who had received a diagnosis of ODD (Fig 2). Of these, 10 eyes were excluded for having no ODD, and 28 eyes were excluded because of ODD-associated anterior ischemic optic neuropathy or other ODD-associated complications. Three hundred twenty-one eyes were included for further analysis. Age, sex, and the presence of PHOMS were noted for all patients (Table 1). The BMO and PHOMS volume were measured and calculated in 112 eyes for which radial scans of the optic nerve were available (Table 1). Sixty-three percent of the patients were female, and 94% of the patients demonstrated bilateral ODD.

**Table 1:** Baseline Characteristics, Peripapillary Hyperreflective Ovoid Masslike Structure Prevalence and Volume in Eyes with Optic Disc Drusen

Variable	Visible Optic Disc Drusen	Buried Optic Disc Drusen	*P* Value	Superficial Optic Disc Drusen	Deep Optic Disc Drusen	*P* Value
No. (%)	209 (68)	99 (32)		147 (46)	174 (54)	
Age (yrs)	45 (26–63)	20 (13–33)	<0.001	39 (22–57)	32 (21–51)	0.31
Male sex	67 (32)	32 (32)	0.9	51 (34)	53 (30)	0.50
BMO (μm)∗	1482 (1411–1550)	1384 (1315–1502)	0.0003	1505 (1418–1665)	1434 (1332–1505)	0.0007
PHOMS	79	72	0.23	84	70	0.002
PHOMS volume (mm^3^)∗	0.25 (0.14–0.41)	0.33 (0.12–0.58)	0.40	0.29 (0.19–0.54)	0.27 (0.11–0.43)	0.36

BMO = Bruch’s membrane opening; ODD = optic disc drusen; PHOMS = peripapillary hyperreflective ovoid mass-like structure(s).

Data are presented as no. (%) or median (interquartile range [IQR]). Patient characteristics are presented, including PHOMS prevalence and volume in patients with visible and buried optic disc drusen (ODD), based on fundus photography, and superficial or deep ODD, based on OCT.

∗ Based on 109 eyes with radial scans and fundus photographs available.

**Figure 3:**
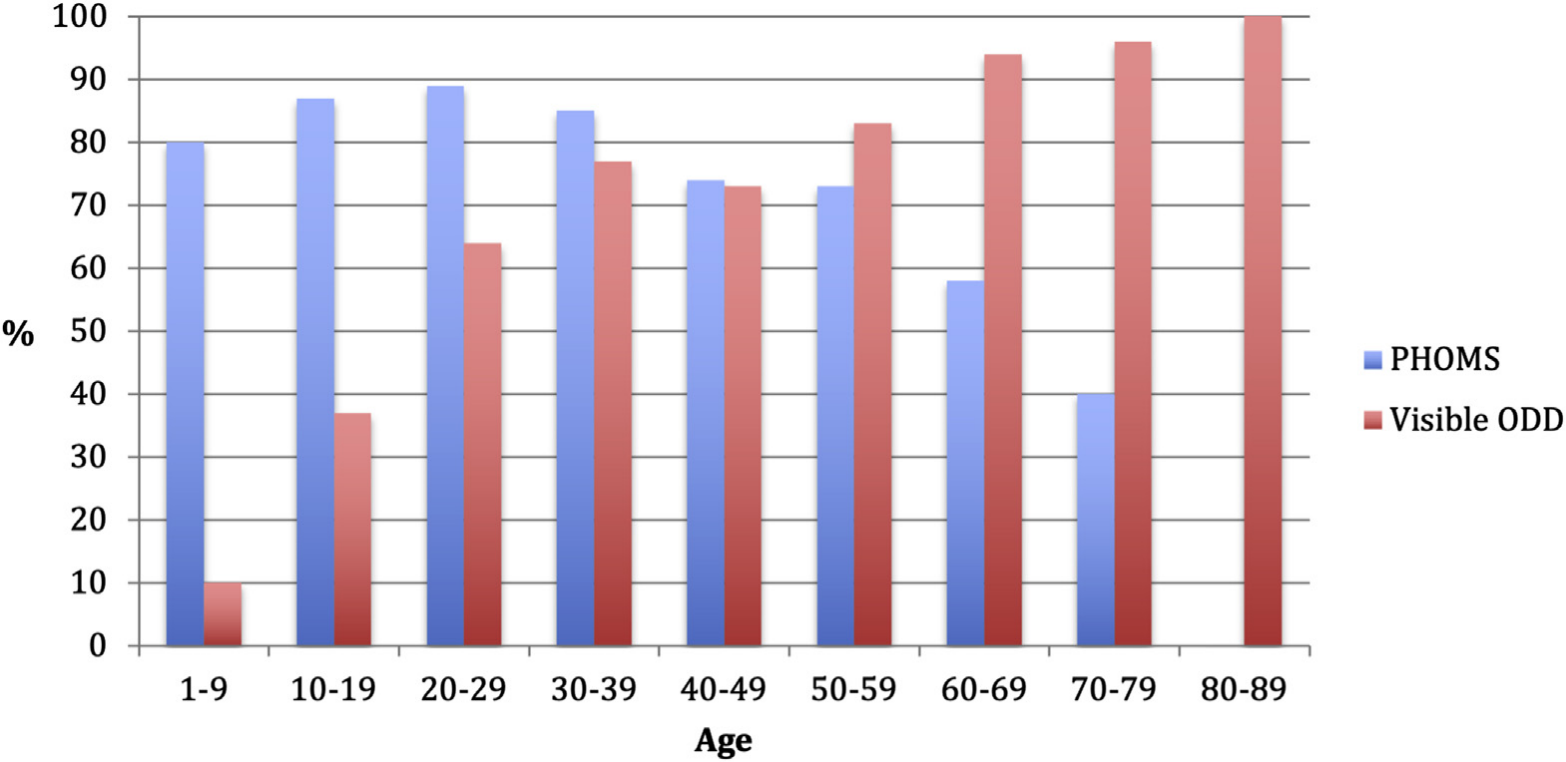
Bar graph showing peripapillary hyperreflective ovoid masslike structure(s) (PHOMS) and visible optic disc drusen (ODD). The prevalence of PHOMS (blue) decreases with age, whereas the ophthalmoscopic visibility of ODD (red) increases with age.

Seventy-six percent of the eyes demonstrated PHOMS, with a median volume of 0.27 mm^3^ (interquartile range [IQR], 0.13–0.49 mm^3^). The presence of PHOMS decreased with age (Fig 3). Of the patients who demonstrated PHOMS, 80% were in first decade of life, 87% were in the second decade of life, 89% were in the third decade of life, 85% were in the fourth decade of life, 74% were in the fifth decade of life, 73% were in the sixth decade of life, 58% were in the seventh decade of life, 40% were in the eighth decade of life, and 0% were in the ninth decade of life.

The visibility of ODD increased with age (Fig 3). Of the patients who demonstrated visible ODD, 10% were in the first decade of life, 37% were in the second decade of life, 64% were in the third decade of life, 77% were in the fourth decade of life, 73% were in the fifth decade of life, 83% were in the sixth decade of life, 94% were in the seventh decade of life, 96% were in the eighth of life, and 100% were in the ninth decade of life.

**Figure 4:**
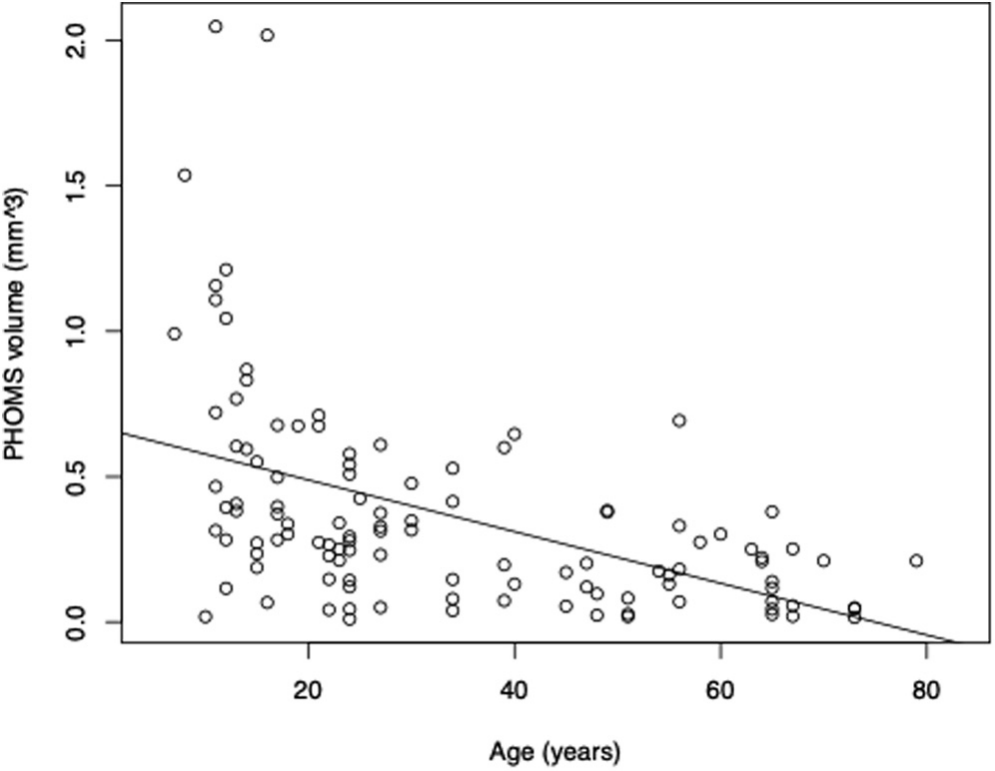
Scatterplot showing peripapillary hyperreflective ovoid masslike structure(s) (PHOMS) volume and age. The volume of PHOMS decreases significantly with age (*P* < 0.05). Based on 112 eyes with radial scans available.

**Figure 5:**
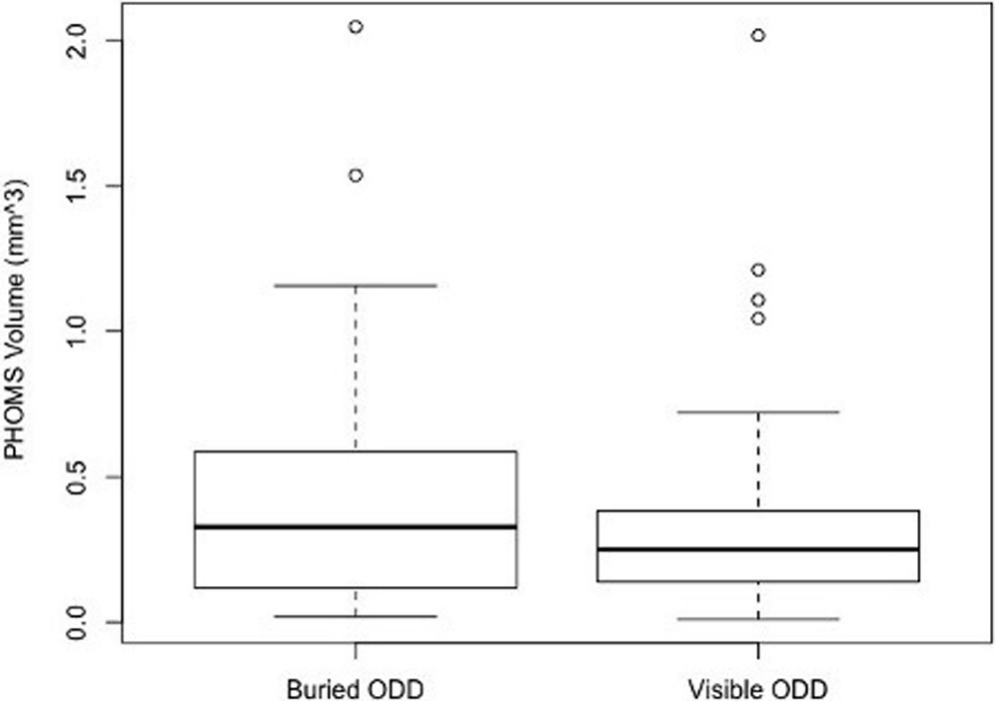
Box-and-whisker plot showing peripapillary hyperreflective ovoid masslike structure(s) (PHOMS) volume in patients with buried and visible optic disc drusen (ODD). Patients with buried ODD harbor larger PHOMS (median, 0.33 mm^3^) compared with those with visible ODD (median, 0.25 mm^3^). The squares represent the interquartile range (IQR), and the lines represent minimum (quartile 1 – 1.5 × IQR) and maximum (quartile 3 + 1.5 × IQR). The circles represent outliers.

The volume of PHOMS ranged from 0.01 to 2.05 mm^3^ and decreased significantly with age (*P* < 0.05) at a rate of 0.009 mm^3^/year (Fig 4). The median volume of PHOMS was 0.27 mm^3^ (IQR, 0.13–0.49 mm^3^). The volume of PHOMS was not related to the diameter of BMO in general, in subdivided age groups, buried ODD, or visible ODD. Based on fundus photography, which was available for 308 eyes, we categorized ODD as either visible (n = 209) or buried (n = 99) and performed subgroup analyses (Table 1). The median volume of PHOMS in patients with buried ODD was 0.33 mm^3^ (IQR, 0.12–0.58 mm^3^). The median volume of PHOMS in patients with visible ODD was 0.25 mm^3^ (IQR, 0.14–0.41 mm^3^; Fig 5).

Based on OCT, which was available for 321 eyes, we recategorized ODD as superficial (n = 147) or deep (n = 174; Table 1). The frequency of PHOMS in patients with superficial ODD was 84%, and the frequency of PHOMS in patients with deep ODD was 70% (*P* = 0.002). Of the 209 eyes with visible ODD, 118 eyes (56%) showed superficial ODD and 91 eyes (44%) showed deep ODD (Table 2). Of the 99 eyes with buried ODD, 23 eyes (23%) showed superficial ODD and 76 eyes (77%) showed deep ODD (Table 2).

**Table 2:** Distribution of OCT-Determined Superficial and Deep Optic Disc Drusen in Eyes with Fundus Photography-Determined Visible or Buried Optic Disc Drusen

	Superficial Optic Disc Drusen	Deep Optic Disc Drusen
Visible ODD	118 (56)	91 (44)
Buried ODD	23 (23)	76 (77)

ODD = optic disc drusen.

Data are presented as no. (%).

**Figure 6:**
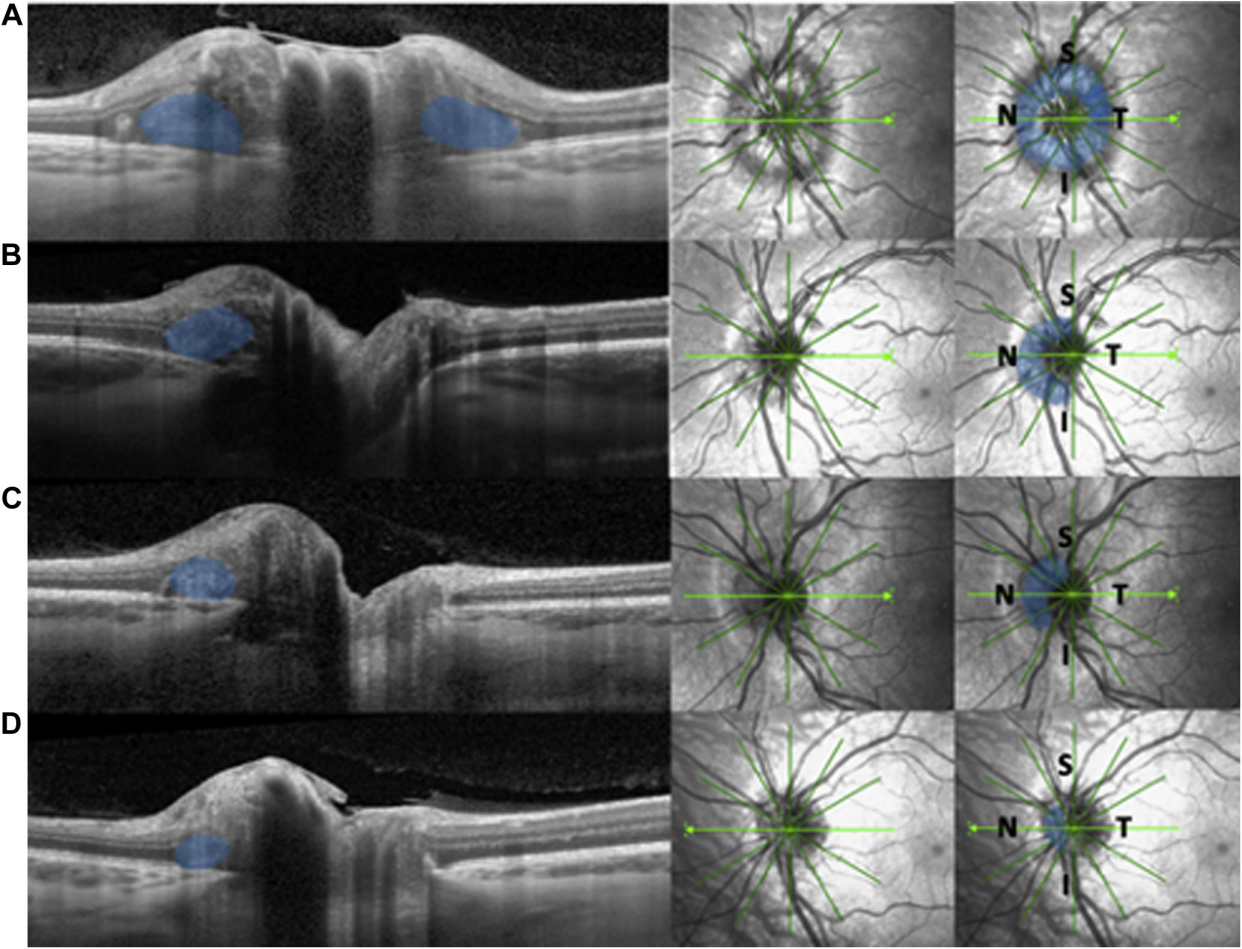
OCT scans demonstrating the NSIT rule of peripapillary hyperreflective ovoid masslike structures (PHOMS): PHOMS are seen most frequently in the nasal peripapillary region, followed by the superior, then the inferior, and finally the temporal peripapillary region. The figure shows 4 different patients with PHOMS extending around Bruch’s membrane opening (BMO) in varying degrees. When PHOMS partially encircle BMO, they are commonly located in the nasal peripapillary areas with varying involvement of the inferior and superior section, whereas superior involvement is seen most frequently. **A**, A PHOMS encircles the BMO in a complete torus. **B**, The PHOMS are located in the nasal, superior, and inferior regions. **C**, The PHOMS is located in the nasal and superior regions. **D**, The PHOMS is located in the nasal region.

The median volume of PHOMS in patients with superficial ODD was 0.29 mm^3^ (IQR, 0.19–0.54 mm^3^). The median volume of PHOMS in patients with deep ODD was 0.27 mm^3^ (IQR, 0.11–0.43 mm^3^). We found that the prevalence of PHOMS was higher in the nasal and superior peripapillary sections, followed by the inferior and temporal sections: nasal, 87.5%; superior, 78.5%; inferior, 67%; and temporal, 63.5%, constituting an NSIT (nasal, superior, inferior, temporal) rule of PHOMS (Fig 6).

## Discussion

Because a PHOMS encircles the optic disc in 3 dimensions, like a torus (full or partial), the true size of PHOMS must be measured as a toroidal volume and not simply as a 2-dimensional cross-sectional area. Based on our review of PubMed/Medline, EMBASE, and Web of Science Core Collection literature databases, this study is the first to assess the volume of PHOMS quantitatively.

Buried ODD are more prevalent in children and younger individuals,^15^ and not surprisingly, we found that the visibility of ODD on fundus photography increases with age. The age-related increased ODD visibility was previously thought to be the result of drusen growth, increase in drusen number, drusen migration, or age-related thinning.^16^ However, our results do not support the theory of ODD migration because we did not find a significant age difference between superficial and deep ODD. A recent long-term OCT follow-up study in children with ODD also concluded that ODD enlarged or developed only in areas with hyperreflective lines, but no ODD migration was seen.^17^ An interesting finding, warranting further study, was that almost half of the eyes with visible ODD actually harbored predominantly deep ODD and that almost 1 of 4 eyes with buried ODD harbored predominantly superficially located ODD.

We showed that not only the prevalence of PHOMS (after 30 years of age), but also their volume, decreases with age. Peripapillary hyperreflective ovoid masslike structures have been shown to correspond histopathologically to herniation of optic nerve fibers from axoplasmic stasis,^13^ and ODD-associated axoplasmic stasis occurs when optic nerve fibers bulge over the rim of BMO, which results in a lateral bulge.^12^^,^^18^ One possible explanation for the negative correlation between age and PHOMS is that, with age-related loss of axons in the optic nerve, neighboring neurons in the scleral canal are decompressed, thereby permitting improved axoplasmic flow, decreased axoplasmic stasis, and less chance of PHOMS formation. The age-related loss of axons could also explain why ODD became more visible with age. Our finding that superficial ODD more frequently cause PHOMS could be explained by the fact that when we graded ODD as being mainly superficial, these were more often large involving areas above and below BMO, whereas ODD graded as being mainly deep more often involved only smaller ODD located only beneath BMO. In future research, a quantitative analysis of the association of PHOMS volume and ODD volume and anatomic location in the optic nerve head would be informative.

We found that the BMO diameter was larger in eyes with visible ODD compared with eyes with buried ODD, which is consistent with a previous study.^19^ Bruch’s membrane opening diameter was also larger in eyes with superficial ODD compared with eyes with deep ODD. It has been suggested that large ODD may extend beyond the margins of BMO and may displace the surrounding structures and lead to overestimation of the BMO diameter or that masking from overlying ODD may cause larger BMO measures.^20^

It has been suggested that patients with a small-diameter BMO demonstrate larger PHOMS.^21^ We did not find a correlation between the diameter of BMO and the volume of PHOMS in patients with either buried or visible ODD. Therefore, the BMO diameter may not significantly affect the formation and volume of PHOMS. However, PHOMS are a common finding in children with myopic or tilted disc, primarily in the nasal peripapillary section.^7^^,^^8^ The tendency for PHOMS to develop in myopic or tilted discs may arise through a combination of structural mechanisms that contribute to axoplasmic stasis, including a protrusion of Bruch’s membrane, the nasal dragging of lamina cribrosa, and the stretching of the temporal sclera;^7^^,^^22^^,^^23^ therefore, other aspects of the anatomic features of the BMO besides its diameter may play a role in the pathogenesis of PHOMS.

A recent review by Fraser et al^12^ categorized PHOMS as: (1) disc edema-associated PHOMS, (2) ODD-associated PHOMS, and (3) anomalous disc-associated PHOMS. The histopathologic features of the optic nerve head are not identical in the 3 categories; therefore, the different conditions and the pathogenesis of PHOMS may not be the same. All 3 subtypes of PHOMS are associated histologically with features of axoplasmic stasis and an S-shaped peripapillary bulge of enlarged, vacuolated nerve fibers over the rim of BMO; however, in disc edema-associated PHOMS, these distended nerve fibers are also accompanied by small dilated venules and capillaries and extravasated interstitial fluid. In ODD-associated PHOMS, such diffuse interstitial fluid extravasation is not seen, but focal compression of nerve fibers by ODD is, and electron microscopy of the nerve fibers may show needles of embedded calcium crystals within the distressed axolemma and mitochondria. Anomalous disc-associated PHOMS may show a variety of different histologic features, depending on cause.^12^ Lorentzen^24^ revealed that ODD are most frequently located in the nasal part of the optic nerve head. In this study, we showed that when a PHOMS does not encircle the disc fully, it is most predominant in the nasal peripapillary region. This strengthens the theory of the ODD-induced axonal compression as a cause of overlying PHOMS.

From a clinical standpoint, it is important to emphasize the nonspecific nature of PHOMS and that PHOMS should not be used qualitatively to differentiate true optic disc edema from other entities such as ODD. When evaluating a patient, the presence of PHOMS indicates only some form of axoplasmic stasis, as may be seen with ODD, optic disc edema, or a crowded or anomalous optic disc. It remains to be elucidated whether PHOMS are an independent risk factor for visual field defects, loss of retinal ganglion cells, and thinning of the peripapillary retinal nerve fibers in both acute and chronic optic neuropathies. In a recent study of 74 eyes with nonarteritic anterior ischemic optic neuropathy (NAAION) in patients younger than 50 years at time of diagnosis, 51% had ODD, whereas PHOMS were seen in 54% of eyes with NAAION and ODD, but in only 28% of those with NAAION, but no ODD.^25^ The surprisingly high prevalence of ODD (and hence PHOMS) in patients with NAAION could indicate an independent role of PHOMS in the development of the compartment syndrome thought to underpin the pathophysiologic characteristics of NAAION. Furthermore, the method for PHOMS volume calculation presented in the study is reproducible, does not require specialized image segmentation software, and is applicable to future longitudinal follow-up studies examining how PHOMS evolve with age in patients with more chronic optic neuropathies.

## References

[1] Spaide R.F., Koizumi H., Pozzoni M.C. (2008). Enhanced depth imaging spectral-domain optical coherence tomography. Am J Ophthalmol.

[2] Fraser J.A., Hamann S.A. (2021). 360-degree peripapillary hyper-reflective ovoid mass-like structure (PHOMS). Can J Ophthalmol.

[3] Malmqvist L., Bursztyn L., Costello F. (2018). Peripapillary hyperreflective ovoid mass-like structures: is it optic disc drusen or not?. Response. J Neuroophthalmol.

[4] Malmqvist L., Bursztyn L., Costello F. (2018). The Optic Disc Drusen Studies Consortium recommendations for diagnosis of optic disc drusen using optical coherence tomography. J Neuroophthalmol.

[5] Traber G.L., Weber K.P., Sabah M. (2017). Enhanced depth imaging optical coherence tomography of optic nerve head drusen: a comparison of cases with and without visual field loss. Ophthalmology.

[6] Teixeira F.J., Marques R.E., Mano S.S. (2020). Optic disc drusen in children: morphologic features using EDI-OCT. Eye (Lond).

[7] Pichi F., Romano S., Villani E. (2014). Spectral-domain optical coherence tomography findings in pediatric tilted disc syndrome. Graefes Arch Clin Exp Ophthalmol.

[8] Lyu I.J., Park K.A., Oh S.Y. (2020). Association between myopia and peripapillary hyperreflective ovoid mass-like structures in children. Sci Rep.

[9] Petzold A., Coric D., Balk L.J. (2020). Longitudinal development of peripapillary hyper-reflective ovoid masslike structures suggests a novel pathological pathway in multiple sclerosis. Ann Neurol.

[10] Malmqvist L., Fraser C., Fraser J.A. (2017). RE: Traber et al.: enhanced depth imaging optical coherence tomography of optic nerve head drusen: a comparison of cases with and without visual field loss (*Ophthalmology*. 2017;124:66–73). Ophthalmology.

[11] Fraser J.A., Rueløkke L.L., Malmqvist L., Hamann S. (2021). Prevalence of optic disc drusen in young patients with nonarteritic anterior ischemic optic neuropathy: a 10-year retrospective study. J Neuroophthalmol.

[12] Fraser J.A., Sibony P.A., Petzold A. (2021). Peripapillary hyper-reflective ovoid mass-like structure (PHOMS): an optical coherence tomography marker of axoplasmic stasis in the optic nerve head. J Neuroophthalmol.

[13] Skougaard M., Heegaard S., Malmqvist L., Hamann S. (2020). Prevalence and histopathological signatures of optic disc drusen based on microscopy of 1713 enucleated eyes. Acta Ophthalmol.

[14] Sung K.R., Wollstein G., Bilonick R.A. (2009). Effects of age on optical coherence tomography measurements of healthy retinal nerve fiber layer, macula, and optic nerve head. Ophthalmology.

[15] Allegrini D., Pagano L., Ferrara M. (2020). Optic disc drusen: a systematic review: up-to-date and future perspective. Int Ophthalmol.

[16] Hamann S., Malmqvist L., Costello F. (2018). Optic disc drusen: understanding an old problem from a new perspective. Acta Ophthalmol.

[17] Malmqvist L., Li X.Q., Eckmann C.L. (2018). Optic disc drusen in children: the Copenhagen Child Cohort 2000 Eye Study. J Neuroophthalmol.

[18] Jonas J.B., Gusek G.C., Guggenmoos-Holzmann I., Naumann G.O. (1987). Optic nerve head drusen associated with abnormally small optic discs. Int Ophthalmol.

[19] Lee K.M., Woo S.J., Hwang J.M. (2013). Morphologic characteristics of optic nerve head drusen on spectral-domain optical coherence tomography. Am J Ophthalmol.

[20] Floyd M.S., Katz B.J., Digre K.B. (2005). Measurement of the scleral canal using optical coherence tomography in patients with optic nerve drusen. Am J Ophthalmol.

[21] Ahn Y.J., Park Y.Y., Shin S.Y. (2021 Mar 17). Peripapillary hyperreflective ovoid mass-like structures (PHOMS) in children. Eye (Lond).

[22] Kim T.W., Kim M., Weinreb R.N. (2012). Optic disc change with incipient myopia of childhood. Ophthalmology.

[23] Lee K.M., Choung H.K., Kim M. (2018). Positional change of optic nerve head vasculature during axial elongation as evidence of lamina cribrosa shifting: Boramae Myopia Cohort Study report 2. Ophthalmology.

[24] Lorentzen S.E. (1966). Drusen of the optic disk. A clinical and genetic study. Acta Ophthalmol (Copenh).

[25] Hamann S., Malmqvist L., Wegener M. (2020). Young adults with anterior ischemic optic neuropathy: a multicenter optic disc drusen study. Am J Ophthalmol.

